# Sestrins at the crossroad between stress and aging

**DOI:** 10.18632/aging.100157

**Published:** 2010-06-13

**Authors:** Jun Hee Lee, Rolf Bodmer, Ethan Bier, Michael Karin

**Affiliations:** ^1^ Laboratory of Gene Regulation and Signal Transduction, Departments of Pharmacology and Pathology, School of Medicine, University of California San Diego (UCSD), La Jolla, CA 92093-0723, USA; ^2^Development and Aging Program, Neuroscience, Aging and Stem Cell Research Center, Sanford-Burnham Medical Research Institute, La Jolla, CA 92037, USA; ^3^ Section of Cell and Developmental Biology, UCSD, La Jolla, CA 92093-0349, USA

**Keywords:** Sestrin, TOR, autophagy, stress, aging, hormesis

## Abstract

Sestrins are a family of stress-inducible proteins that can function as antioxidants and as inhibitors of target of rapamycin complex 1. In this research perspective, we discuss the possible roles of Sestrins in diverse stress-induced patho-physiological contexts that can result in premature aging and age-related diseases. We suggest that Sestrins provide critical feedback regulation that adjust metabolic and stress responses to different environmental cues and evolutionary constraints.

The discovery of Sestrins as p53 targets [1,2] suggested that these proteins are stress-inducible because they protect cells against various insults [2]. Sestrins have dual biochemical functions, as antioxidants that control the activity of peroxiredoxins which scavenge reactive oxygen species (ROS) [3] and as inhibitors of target of rapamycin complex 1 (TORC1) signaling [4,5]. Both ROS accumulation [6] and TORC1 activation [7,8] are associated with accelerated aging and development of age-associated pathologies in diverse organs and organisms, implicating Sestrins as anti-aging agents. Conversely, reduction of ROS accumulation with antioxidants or as a result of TORC1 inhibition [7-12] causes an extension of life span as well as health span. Indeed, we recently confirmed Drosophila Sestrin (dSesn) prevents age-associated pathologies including fat accumulation and cardiac and skeletal muscle degeneration by providing a feedback loop that prevents excessive TORC1 activation and ROS accumulation [4]. In this research perspective, we discuss the possible roles of Sestrins in diverse stress-induced patho-physiological contexts that can result in premature aging and age-related diseases. We suggest that Sestrins provide critical feedback regulation that adjust metabolic and stress responses to different environmental cues and evolutionary constraints.

## DNA damage

**Figure 1.**
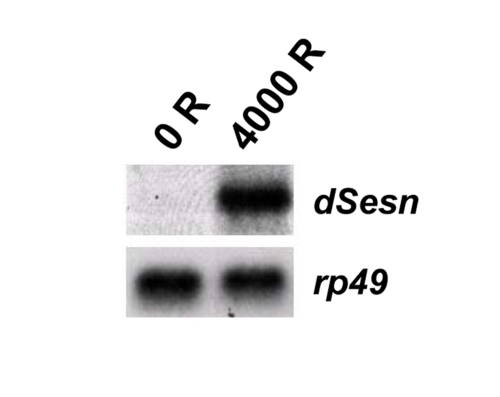
Induction of *dSesn* after DNA damage. First instar fly larvae were challenged with 4000 rads (R) of gamma radiation, and RNA was extracted after 4 hrs. Northern blot analysis revealed that *dSesn* mRNA is highly induced upon irradiation. *rp49* mRNA was used as a loading control.

Chronic exposure to genotoxic stress is known to accelerate aging, and mutations that disrupt proper DNA damage responses and interfere with DNA damage repair are associated with premature aging in humans [13]. Many studies have established that genotoxic stresses can inhibit protein and lipid synthesis, and that these coordinated responses may be essential for survival because reducing energy expenditure on macromolecule biosynthesis can divert scarce resources to the repair of damaged DNA [14]. Sestrins, as DNA damage-inducible proteins, may play a critical role in this process [15]. Both mammalian Sesn1 and Sesn2 are induced upon DNA damage in response to activation of p53 [1,2], and dSesn is also induced upon radiation-induced DNA damage (Figure 1). Increased Sestrin abundance potentiates the activity of AMP-activated protein kinase (AMPK), thereby diminishing TORC1 activity [5]. Reduced TORC1 activity inhibits anabolic pathway including protein and lipid synthesis [16,17]. Shutdown of TORC1-dependent anabolism upon genotoxic stress is likely to be important for minimizing new protein and membrane synthesis and use the energy that was thus saved to promote DNA repair. Therefore, DNA damage-dependent induction of Sestrins may minimize the detrimental effects of DNA damage that contribute to accelerated aging and various pathologies that are associated with premature aging.

Age-dependent accumulation of DNA damage can lead to cancer [13], one of the leading causes of mortality worldwide [18]. Therefore, Sestrin induction in response to DNA damage [1,2] may contribute to the many tumor suppressor functions carried out by p53 [19]. In addition to inhibiting cell proliferation and promoting the death of cells with excessive DNA damage, p53 was recently found to inhibit TORC1 [14] and to suppress cell growth as well as cellular and organismal senescence [19-21]. We found that Sesn1 and/or Sesn2 are critical mediators of p53-induced TORC1 inhibition in cultured cells and in mouse liver [5]. In addition, Sestrins can suppress the growth of some cancer cell lines [2] and loss of Sesn2 makes immortalized cells more susceptible to oncogenic transformation [5]. The SESN1 (6q21) and SESN2 (1p35) loci are frequently deleted in a variety of human cancers [1,22,23], implicating loss of Sestrins in tumor progression and suggesting that Sestrin-dependent inhibition of TORC1 is critical for suppressing tumorigenesis spurred by age-dependent accumulation of damaged DNA.

## Oxidative stress

Oxidative stress not only can interfere with the proper flow of genomic information by oxidizing DNA and RNA, but also can damage other macromolecules such as proteins and lipids [6]. Accumulation of oxidative macromolecular damage causes cellular senescence, tissue aging and reduced life span [6], as well as neurodegeneration [24] and metabolic disorders [25], which are diseases associated with aging. Amongst the organelles that are affected by oxidative stress, mitochondria appear to be the most sensitive [6,26]. Moreover, damaged mitochondria are a major source of ROS [6], which escalates oxidative damage in stressed cells. Extensive mitochondrial dysfunction causes cell death, and in some cases can lead to neuronal or muscular degeneration [6,24,27]. To prevent the detrimental consequences of mitochondrial dysfunction, cells eliminate damaged mitochondria through an autophagic process, called mitophagy [28,29].

Sestrins are transcriptionally induced upon oxidative stress [2], and are important for cell survival under oxidative stress [2,3,30,31]. Sestrins can function as oxidoreductases in vitro and in vivo that lead to the reactivation of peroxiredoxin [3], and may be involved in reducing oxidative stress [30-32] by scavenging ROS and/or regenerating reduced peroxiredoxin [3]. Independent of their oxidoreductase activity, Sestrins induce autophagy by inhibition of TORC1 [5,33,34]. Enhanced autophagy results in more efficient elimination of ROS-producing damaged mitochondria in stressed cells [28,29]. Sestrin-induced activation of AMPK and inhibition of TORC1 can also reduce ROS production by increasing the efficiency of mitochondrial respiration [11,12]. Therefore, Sestrins have a key role in maintaining cellular integrity and homeostasis during oxidative insults.

## Hypoxia

Hypoxia is another environmental stimulus that can induce Sestrin gene transcription [2]. Sestrins protect cells from apoptosis during hypoxic conditions [2], and Sestrin-induced shutdown of TORC1 signaling can reduce cellular energy consumption that is likely to improve adaptation to hypoxic conditions. Sestrin-stimulated autophagy can provide an additional energy source and at the same time eliminate dysfunctional mitochondria generated by inefficient respiration under low oxygen tension.

Ischemic injury to heart muscles and neurons, which is caused by hypoxia, is one of the major causes of death in elderly individuals [18]. In an experimental model of acute stroke, Sesn2 was shown to be highly induced upon hypoxic injury [2], suggesting that Sesn2 may exert its neuroprotective role during stroke. In the heart, hypoxic injury and re-oxygenation cause bursts of ROS production, which can cause irreversible damage to the heart muscle, resulting in cardiac arrhythmia and heart failure [35,36]. In the Drosophila heart, both aging [37-41] and hypoxia [39,42,43] cause cardiac dysfunction, and activation of TORC1 pathway aggravates or accelerates age-associated arrhythmicity and heart failure [44]. Loss of dSesn function results in a similar cardiac arrhythmicity [4], suggesting a cardio-protective function of Sestrin in restraining TORC1 activity. Thus Sestrin expression retards the appearance of age-associated cardiac pathologies, as was previously observed in response to genetic reduction of TORC1 function [10,44].

Hypoxic preconditioning can protect both heart and neuronal cells from severe ischemic injury-induced cellular damage [36,45], but the underlying mechanisms were not elucidated. Induction of autophagy upon preconditioning was suggested to be required for protection of heart and neuronal cells from hypoxic insults [36,46]. An intriguing possibility to investigate therefore is whether hypoxic preconditioning induces Sestrin to increase the level of autophagy that is required for the prevention of serious heart attacks and neurological strokes.

**Figure 2.**
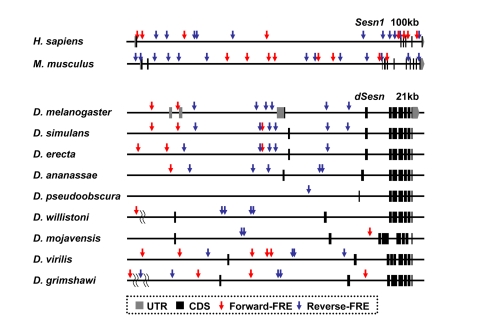
FoxO binding sites in the *Sestrin* locus vary among the species. Genomic organization and location of FoxO response elements (FRE, GTAAACAA [57]) in the *Sesn1* locus of human and mouse [58] and the *dSesn* locus of various *Drosophila* species [59,60] with indicated genomic span. Gray boxes indicate untranslated exons (UTR), and black boxes indicate coding exons (CDS). For *Drosophila* species other than *D. melanogaster*, untranslated regions of *dSesn* mRNAs are currently unknown. Forward FREs are indicated by red arrows and reverse FREs by blue arrows.

## An evolvable link to the environment

In addition to the important role that Sestrins play in mediating essential environmental inputs into metabolic regulation, these molecules may also play central roles in responding to other environmental cues such as nutrient supply, hydration status, temperature, chemical damage, and reproductive signals. One might speculate that because these various cues would have different impacts on different organisms, Sestrin genes should have evolved complex cis-regulatory regions to place them under distinct regulatory controls that vary from one organism to another. Indeed, our analysis of Sestrin genomic loci revealed that these sequences are rapidly evolving among the various Drosophila species. For example, the disposition and number of FoxO binding sites in the Sestrin locus vary significantly among species (Figure 2). Comparative studies of cis-regulatory sequences between Drosophila species by swapping these sequences using recombineering techniques may shed light on mechanisms by which selective pressures sculpt the stress response during evolution.

**Figure 3.**
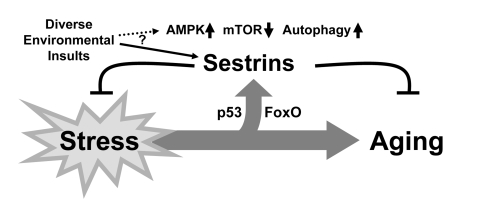
Schematic diagram hypothesizing the role of Sestrin as a brake of stress-accelerated aging processes. Various environmental insults increase expression of Sestrins to affect Sestrin-mediated regulation of AMPK-TORC1 signaling.

## Concluding remarks

In this research perspective, we briefly reviewed how Sestrins may act as suppressors of aging that are responsive to stressful stimuli and insults that can accelerate the aging process. By activating AMPK and inhibiting TORC1, Sestrins can reprogram cells to adapt to stressful conditions by attenuating anabolism and enhancing autophagic catabolism. Sestrins can suppress oxidative damage by acting as either antioxidants, inducers of autophagy that eliminate ROS-producing dysfunctioning mitochondria, or suppressors of ROS-producing mitochondrial metabolism. Therefore, we can view Sestrins as physiological brakes that can attenuate stress-dependent acceleration of aging (Figure 3).

In addition, it is worth noting that Sestrins are also expressed under normal unstressed conditions [1,2,4], and that dSesn knockout mutants show accelerated aging phenotypes even in the absence of any environmental stress [4]. Therefore, it is possible that Sestrins provide a baseline protective function that reduces the damage from physiological insults that are unavoidable consequences of basic life processes such as oxidative respiration and DNA replication.

Over-nutrition and obesity can elevate the incidence and frequency of physiological insults by stimulating TORC1 activity [47], which in turn accelerates anabolic metabolism [16,17]. Hyperactive TORC1 can enhance the accumulation of unfolded and aggregated proteins [48] and ROS [4,12,49], leading to stress responses that increase Sestrin expression, thereby activating negative feedback loops that readjust AMPK and TORC1 activity [4]. Therefore, Sestrins may also function as metabolic brakes that attenuate the pathological consequences of over-nutrition and its associated TORC1 hyperactivity [50]. An interesting human evolutionary question in this regard is whether regulation of Sestrin induction has been weakened in populations subject to frequent starvation conditions since such populations have been shown to be particularly at risk for obesity, presumably as a result of their altered response to nutrient cues [51,52].

Although environmental stress generally accelerates aging, it should be considered that exposure to a low level of stress or stress adaptation can actually be beneficial for the organism, increasing life span and preventing age-associated degenerative diseases [53-56]. The beneficial effect of low-level stress exposure, referred to as hormesis, was observed in both experimental animal models and human subjects [53-56], but no decisive molecular explanation has been provided to explain this paradoxical phenomenon. Given that Sestrins are upregulated in response to a variety of stresses [1,2], it will be interesting to investigate whether stress-induced Sestrins are also mediators of the hormetic effect.

In summary, we suggest that Sestrins are uniquely poised at the crossroads between stress and aging to adjust the metabolic timbre of an organism to meet its needs under normal conditions and to respond to predictable forms of environmental stress.  Future experiments should shed light on the specific mechanisms by which the various effector functions of the Sestrins are achieved.
